# A Method of Making Inlays for Cavities Difficult of Access

**Published:** 1903-04-15

**Authors:** J. S. M'Curdy


### A METHOD OF MAKING INLAYS FOR CAVITIES DIFFICULT OF ACCESS.

BY DR. J. S. M'CURDY.

Prepare the cavity cup-shape with well-defined edges. When prepared, vaseline the tooth and cavity and take an impression with pink sheet gutta-percha. Fill this impression with amalgam, and when thoroughly hard swage platinum foil 1-1000 of an inch in thickness between impression and mold thus made, forming an accurate fitting matrix in which to bake the porcelain inlay. This method is particularly applicable to those teeth where the platinum can not be easily burnished into the cavity direct.

